# Correction: An epidemiological study of gastrointestinal nematode and *Eimeria* coccidia infections in different populations of Kazakh sheep

**DOI:** 10.1371/journal.pone.0272760

**Published:** 2022-08-03

**Authors:** Xiaofei Yan, Mingjun Liu, Sangang He, Ting Tong, Yiyong Liu, Keqi Ding, Haifeng Deng, Peiming Wang

The email address for the corresponding author is incorrect. The correct email address for Mingjun Liu is: xjlmj2006@126.com.

An affiliation is missing for the corresponding author. Mingjun Liu is affiliated with #1 and #3. The correct affiliations are:

**1** College of Animal Science and Technology, Shihezi University, Shihezi, Xinjiang Province, China, **3** Institute of Biotechnology, Xinjiang Academy of Animal Science, Key Laboratory of Genetic Breeding and Reproduction of Herbivorous Livestock of Ministry of Agriculture, Xinjiang Key Laboratory of Animal Biotechnology,Urumqi, Xinjiang Province, China
